# Treatment of the Irritable Bladder in Women

**Published:** 1881-11

**Authors:** J. H. Etheridge


### ARTICLE II.

TREATMENT OF THE IRRITABLE BLADDER IN WOMEN. By J. H. ETHERIDGE, M. D. (Read before the Chicago Gynecological Society, November, 1880.)

A too frequent desire to empty the bladder may be said to constitute what is called "Irritable Bladder." Hyperæsthesia, supersensibility of the viscus is the cause of this malady. It ought not to be confounded with irritability of the bladder, which is a physiological, not a pathological phenomenon. "Irritable Bladder," and "Hyperæsthesia of the Bladder" can be used as synonymous expressions.

The *cause* of irritable bladder determines its treatment. When it is unknown, treatment becomes guesswork. Consequently the physician's first problem of treatment is the determining of the *cause* in each individual case of vesical hyperæsthesia.

CAUSES of irritable bladder may be divided into *intrinsic* and *extrinsic* causes.

The *intrinsic causes* include abnormalities of the urine, consisting of—

1st—Too *limpid urine.*

2nd—Too *concentrated urine.*

3rd—An excess of *uric acid*, as shown by gravel, calculi, and amorphous urates.

4th—*Triple* and *amorphous phosphates*, shown in decomposition of the urine.

5th—*Oxaluria*, and

6th—*Sugar* and *albumen.*

Among intrinsic causes may be included abnormal substances not of urinary origin, which may be enumerated as follows:

7th—*Pus* and *blood* from renal or cystic diseases.

8th—*Feculent matter*, gall-stones, joints of tapeworms, and round worms.

9th—*Hair*, *fat*, *teeth* and *bones* from a fistulous communication with a dermoid cyst.

Other intrinsic causes include—

10th—*Cystitis, acute or chronic.*

11th—*Malignant disease* of the bladder, primary or secondary.

12th—*Polypi.*

13th—*Cysts* and *tubercles.*

14th—*Hypertrophy*, centric or eccentric.

Intrinsic causes may include disorders of the urethra as well as of the bladder, and are thus indicated:

15th—*Urethritis*, acute and chronic.

16th—*Neoplasm.*

17th—*Dilatation of the urethra*, including that of the upper third, and of the whole canal.

18th—*Dislocation of the urethra.*

19th—*Prolapsus of the urethral mucous membrane.*

20th—*Stricture*, and

21st—*Incomplete Fistula.*

The extrinsic causes of Irritable Bladder are numerous, often difficult to define, and are much more common than intrinsic causes. Fully two-thirds of the cases of this disorder arise from extrinsic causes. Presenting uniformly but the one symptom of frequent urination, these causes are infinitely less correctly differentiated by physicians, and consequently are worthy of the closest scrutiny and most careful management, therapeutically and otherwise. Every experienced gynecologist can recall only the many defeats in the treatment of Irritable Bladder from extrinsic causes because of not accurately ascertaining the causes in each case. These causes include:

*a.*—*Oöphoria*, or ovarian, vascular excitement.

*b.*—*Pressure on the bladder or urethra* from the uterus, or from rectal abnormalities, or from intra-pelvic tumors.

*c.*—*Sympathetic irritation* from uterine inflammation.

*d.*—*Malignant disease of the cervix uteri.*

*e.*—*Pregnancy.*

*f.*—*Vaginismus.*

*g.*—*Acute pelvic peritonitis and cellulitis.*

*h.*—*Ascarides.*

*i.*—*Hysteria.*

*k.*—*Mental troubles, fright.*

*l.—Exposure during menstruation.*

*m.—Falls, and blows over the bladder.*

*n.—Masturbation and copulation ;* and

*o.—Malaria.*

The causes herein mentioned are enumerated in extenso, for the purpose of showing that the treatment cannot be the same in all cases of irritable bladder.

The management of cases produced by causes numbered 1 to 5 inclusive, usually involves remedies addressed to nutrition. *Too limpid urine* suggests hysteria, anemia, exposure to cold, mental emotion, in short, diuresis from any cause. Treatment of these conditions involves remedies addressed to the cause whatever it may be.

*Too concentrated urine*, shown by the small amount voided and by high specific gravity, calls for water, simply. Ordinary drinking water, Poland water, Apollinaris water, or any of the simple mineral waters, capable of increasing the amount of water excreted by the kidneys will answer.

*An excess of uric acid* is one of the most common of all of the many causes of irritable bladder. Neglected, it speedily causes a congestion of the mucous membrane of the bladder, and this, in turn, propagates the polyuria and dysuria. The organic changes arising from this congestion are of a sufficiently progressive character to afford a constantly acting cause of irritable bladder. In this way, a bladder thus afflicted, at first, say, three months ago, presents *to-day* an irritable condition arising from the products of chronic congestion, whereas the excess of uric acid starting in motion this pathological train of symptoms ninety days ago, now occupies a very inferior position in the cause to-day. Consequently the majority of cases treated by physicians present not only an excess of uric acid, but they present also a vesical mucous membrane congestion. To treat this condition requires skill and patience. An excess of uric acid indicates a systemic, and, especially, an alimentary defect. This excretion, possessed of another particle of oxygen, becomes urea and ceases to be a pathological product. To supply that oxygen is no easy matter. If the blood be made to carry more oxygen and thus supply the deficiency, we must give remedies to improve the oxygenating power of the blood. For this purpose one quarter to one grain doses of permanganate of potash, thrice daily, will be found useful. This powerful oxydising agent yields up its oxygen in the form of ozone and converts uric acid into urea. Irritable bladder relieved by the permanganate is *relieved* simply—*not cured.* The alkaline carbonates neutralize uric acid excess directly, and indirectly diminish it by their action on the liver. The citrate or carbonate of lithium dissolves uric acid, and is a remedy of undoubted efficacy: (Murchison, Sir Henry Thompson). Colchicum, in small doses, improves the character of the digestive ferments and promotes more perfect digestion, and in this way supplies the liver with better pabulum, thus causing a lessening of the amount of uric acid (Lauder and Brunton). Its action is surprisingly happy in very many cases—but in many other cases it seems to extend the point of tolerance, causing, unexpectedly, a vomiting and violent purging, an effect greatly to be deprecated. Fruit acids are converted into the alkaline carbonates in the blood and become dissolvers of uric acid or diuretics, and in this way are efficient aids in treating the uric acid excess. Many patients will be benefited by a hot lemonade at bedtime, or by eating a lemon before breakfast daily, or by partaking liberally of acid fruit at breakfast and lunch times. The uric acid excess thus relieved can be said to be relieved only—not cured. It is well to use them because they act quickly and satisfactorily. Thus relieved temporarily, the physician can take the necessary time to determine where the ultimate cause of uric acid excess lies and select remedies to cure it. The systemic defect is usually the ultimate cause and will be found in the primary or secondary assimilation. Constipation is almost always a conspicuous symptom in these cases. Its resultant evils include stomachic and intestinal indigestion. From it results a condition of blood poisoned with excretory material, which causes all functions of the abdominal organs to be imperfectly performed. The secreting cells of the liver fail to elaborate their products perfectly, and from this failure arises the uric acid excess. A thorough catharsis temporarily relieves all of these symptoms, and it is an exceedingly unwise thing to do to use powerful purges, because they are of only temporary benefit. The course to pursue is to give a daily laxative, and thus by degrees purify the blood and secure a sustained functional improvement in the organs of primary assimilation. To this end, the daily use of Rakocszy, Hunyadi Janos, Friedericshall, or Victoria mineral water in warm weather; or of aloes, podophyllin, cascara sagrado, compound extract of colocynth, compound liquorice powder, or euonymus, in cold weather, will be useful. To improve the secondary assimilation we can resort to the use of bark and iron, arsenic, strychnia, minute doses of mercury when not contra-indicated, and coca. Of prime importance in these cases is the management of the diet. Farinaceous articles and acid fruits should be largely used, and only as much of the albuminous articles used as can be thoroughly digested.

Irritable bladder arising from *triple and amorphous phosphates* should be treated by treating the systemic condition producing them. They are usually found in diseases of the nerve centers and after great mental application. They generally suggest the use of rest, ergot, galvanism, massage, tonics and improvement of alimentation.

*Oxaluria* should be treated by paying especial attention to the "moral, mental and physical condition, and time must not be wasted in treating a mere symptom:" (Skeene). Strychnia and the mineral acids will yield the best results.

Cases involving *diabetes* or *albuminuria* call for treatment addressed exclusively to these conditions.

Irritable bladder arising from causes enumerated, 7th to 9th, inclusive, must be treated according to indication wholly, which consists in the removal from this viscus of these foreign substances.

Cases involving *cystitis, acute or chronic*, the 10th cause enumerated, are troublesome enough. Some patients will make rapid recoveries. But by far the largest majority of them will prove rebellious. The urine in these cases must be rendered alkaline as speedily as possible. Citrate of potassium, in as large doses as can be borne without causing stomachic distress, is an excellent remedy. The removal of existing constipation by daily laxative doses of mineral water upon arising mornings, is of importance, as it secures a systemic condition favorable to producing urine of minimum acidity, after which smaller doses of the citrate of potassium will suffice to produce alkalinity of the urine. At the same time, restricting the diet to articles calculated to aid in avoiding acid excess in this excretion, should be prescribed. An exclusive milk diet has cured cases of long standing and of great severity. Alkalis, minim doses of tr. cantharides, hourly, twenty grain doses of bromide of ammonium, the solution of bromohydric acid, benzoate of ammonia in buchu, laxatives, proper diet, quietude, will relieve most cases of irritable bladder from *acute* cystitis.

The irritable condition arising from *chronic* cystitis, requires a wider range of remedial measures to meet all cases. Many women, however, are never cured, failure arising from many causes, as lack of pertinacity on the patient's part in submitting to treatment, dyscrasia, failure to apprehend and to remove co-existing disorders which sympathetically propagate the cystitis, etc., etc. Frequent urinalyses are necessary to guide us in the administration of medicines, internally. Acidity is to be modified or abolished by alkalis. Urinary decomposition calls for the sulpho-carbolates, for alyptus globulus, or for salicylate of sodium. Medicated injections into the bladder are all important in chronic cystitis. A long range of remedies is before us to select from. Antiseptic injections include solutions of common salt, potassium chlorate, carbolic acid, salicylic acid, eucalyptol, and the sulpho-carbolates. Astringent and alterative injections embrace silver nitrate, hydrastis canadensis, tannic acid, plumbic acetate, iodoform, sulphate of zinc and potassic iodide. Forcible dilatation of the urethra, self-retaining catheter and cystotomy are resorted to only after other means have failed.

Cases involving cause enumerated 11, will never be relieved permanently. Opiates or chloral may be used.

Polypi must be removed. No remedies can be resorted to in cases of too frequent urinations caused by polypi.

Cysts and tubercles are rare vesical troubles and usually cause irritable bladder. General tonics to abrogate the systemic condition producing them, and opiates to alleviate the irritable condition seem to be the remedies demanded.

Hypertrophy of the bladder, a not uncommon cause of dysuria and polyuria, must have its cause removed. Tumors and cystocele must be treated *secundum artem ;* neuralgia or any functional disorder operating to cause this condition, must be considered and treated before the irritable condition can be removed.

Urethritis, acute or chronic, is a most troublesome condition to remove. The acute form generally calls for the same sort of management accorded to gonorrhœa in the male. The subacute or chronic form calls for injections, alterative and astringent. The utmost patience is necessary to *cure* cases of chronic urethritis.

Neoplasms demand surgical treatment. The general condition of the patient's health can be greatly improved by medication. Tumors or pressure in any way retarding venous circulation from the pelvis demands attention and removal if possible. Removal of the neoplasm will relieve the patient of the irritable bladder.

Cases involving causes numbering from 17 to 21 inclusive, suggest their remedies, which are surgical or mechanical.

The treatment of cases of irritable bladder produced by extrinsic causes is included in the management of those causes. The fact cannot be emphasized too much, that the most troublesome aspect of the treatment of irritable bladder in women is the ascertaining of the *cause.* The cause being determined, the principles of treatment are usually very simple.

